# Supplementary material for: Early dynamics of chronic myeloid leukemia on nilotinib predicts deep molecular response
Source: NPJ Syst Biol Appl. 2022 Oct 13;8:39. doi: 10.1038/s41540-022-00248-3 (PMC9561725; doi:10.1038/s41540-022-00248-3)
Supplement: Supplementary file 1 — Supplementary Materials [file 41540_2022_248_MOESM1_ESM.pdf]

Supplementary Materials for

Early Dynamics of Chronic Myeloid Leukemia on Nilotinib Predicts Deep Molecular Response

Yuji Okamoto[1,2†‡], Mitsuhito Hirano[2‡], Kai Morino[1,3], Masashi K. Kajita[1,4,5,*], Shinji Nakaoka[1,6,7], Mayuko Tsuda[2], Kei-ji Sugimoto[8], Shigehisa Tamaki[9], Junichi Hisatake[10], Hisayuki Yokoyama[11], Tadahiko Igarashi[12], Atsushi Shinagawa[13], Takeaki Sugawara[14], Satoru Hara[15], Kazuhisa Fujikawa[16], Seiichi Shimizu[17], Toshiaki Yujiri[18], Hisashi Wakita[19], Kaichi Nishiwaki[20], Arinobu Tojo[2,21], Kazuyuki Aihara[1,22,*]

Affiliations

[1]Institute of Industrial Science, The University of Tokyo; Tokyo, 153-8505, Japan.

[2]Division of Molecular Therapy, Advanced Clinical Research Center, The Institute of Medical Science, The University of Tokyo; Tokyo, 108-8639, Japan.

[3]Interdisciplinary Graduate School of Engineering Sciences, Kyushu University; Fukuoka, 816-8580, Japan.

[4]Department of Applied Chemistry and Biotechnology, Faculty of Engineering, University of Fukui; Fukui 910-8507, Japan.

[5]Life Science Innovation Center, University of Fukui; Fukui 910-8507, Japan.

*Corresponding author. Email: masashi.k.kajita@gmail.com

[6]Faculty of Advanced Life Science, Hokkaido University; Hokkaido 060-0810, Japan.

[7]PRESTO, Japan Science and Technology Agency; Tokyo 102-0076, Japan.

[8]Division of Hematology, Juntendo University Urayasu Hospital; Chiba 279-0021, Japan.

[9]Department of Hematology, Japanese Red Cross Ise Hospital; Mie 516-8512, Japan.

[10]Department of Hematology, Japanese Red Cross Omori Hospital; Tokyo 143-8527, Japan.

[11]Department of Hematology, National Hospital Organization, Sendai Medical Center; Miyagi 983-8520, Japan.

[12]Divison of Hematology and Oncology, Gunma Cancer Center; Gunma 373-0828, Japan.

[13]Department of Internal Medicine, Hitachi General Hospital; Ibaraki 317-0077, Japan.

[14]Division of Hematology-Oncology, Chiba Cancer Center; Chiba 260-8717, Japan.

[15]Department of Hematology, Chiba Rosai Hospital; Chiba 290-0003, Japan.

[16]Department of Hematology, Chibaken Saiseikai Narashino Hospital; Chiba 275-8580, Japan.

[17]Department of Hematology, Tsuchiura Kyodo General Hospital; Ibaraki 300-0028, Japan.

[18]Third Department of Internal Medicine, Yamaguchi University; Yamaguchi 755-0046, Japan.

[19]Division of Hematology and Oncology, Japanese Red Cross Narita Hospital; Chiba 286-8523, Japan.

[20]Division of Oncology and Hematology, Jikei University Kashiwa Hospital; Chiba 277-8567, Japan.

[21]Institute of Innovation Advancement, Tokyo Medical and Dental University; Tokyo 113-8510, Japan.

[22]International Research Center for Neurointelligence (WPI-IRCN), The University of Tokyo; Tokyo 113-0033, Japan.

*Corresponding author. Email: kaihara@g.ecc.u-tokyo.ac.jp

†The present address: Department of Biomedical Data Intelligence, Graduate School of Medicine, Kyoto University; Kyoto 606-8507, Japan.

‡These authors contributed equally to this work.

This PDF file includes:

Supplementary note 1. EUTOS score-based and ELTS score-based DMR prediction
Supplementary figure 1. Measurement data for CML patients
Supplementary figure 2. Our CML model approximated all measurement data
Supplementary figure 3. Effectiveness of the EUTOS score, ELTS score, and European LeukemiaNet guideline criteria for MR4.0, MR4.5, and CMR
Supplementary figure 4. The classification of MR4.5 and CMR patients required the lower CML cell convergence value than that of MR4.0 patients
Supplementary figure 5. Our prediction method can also be applied to MR4.0, MR4.5 and CMR
Supplementary figure 6. Confusion matrices of DMR prediction based on our prediction method and the criteria of the current scoring systems and guidelines
Supplementary figure 7. Patient characteristics based on the results of the EUTOS score-based DMR prediction
Supplementary figure 8. Patient characteristics based on the results of the ELTS score-based DMR prediction

Other Supplementary Materials for this manuscript include the following:

Supplementary data 1. (separate file)

Supplementary note 1. EUTOS score-based and ELTS score-based DMR prediction

To understand the characteristics of the patients whose deep molecular response (DMR) predictions based on the EUTOS score or the EUTOS long-term survival (ELTS) score failed, we investigated the patient characteristics of the variables to calculate the scores (Supplementary figures 6-8). For readability, here we repeat the definitions of the EUTOS score and the ELTS score, and the variables used in the scores. The two scores are defined as follows:

$$\text{EUTOS score} := 4 \times \text{spleen size below costal margin (cm)} + 7 \times \text{basophil ratio (\%)},$$

$$\text{ELTS score} :=$$

$$0.0025 \times \left(\frac{\text{age in completed years}}{10}\right)^{3}$$

$$+ 0.0615 \times \text{spleen size below costal margin (cm)}$$

$$+ 0.1052 \times \text{blasts in peripheral blood (\%)}$$

$$+ 0.4104 \times \left(\frac{\text{platelet count}}{1000}\right)^{-0.5}.$$

If a patient has a high score, the patient is considered as “high risk” or “intermediate risk”, which corresponds to “prediction as non-DMR” in this study. If a patient has a low score, the patient is considered as “low risk”, which corresponds to “prediction as DMR”. According to the score definition, the scores are monotonically increasing functions of the variables. It means that these scores are designed so that patients with large variable values tend to be predicted as non-DMR.

To investigate the characteristics of the patients whose prediction based on the EUTOS score or ELTS score failed, we classified the N-road study patients into four subsets based on the prediction results. The four subsets are as follows: predicted as true positive (TP), false positive (FP), false negative (FN), and true negative (TN) cases. The numbers of patients in the cases are indicated in

the confusion matrices shown in Supplementary figure 6. In the N-road dataset, compared with our method, the EUTOS score-based prediction tends to classify patients into FP cases, while the ELTS score-based prediction tends to classify patients into FN cases (Supplementary figure 6). Please note that, in the analyzed dataset, no patients were classified as FP cases by the ELTS score-based prediction (Supplementary figure 6 and Supplementary figure 8).

Then we compared the characteristics of the patients in the four subsets based on the average values and the distributions of the variables. The results of the EUTOS score-based prediction and those of the ELTS score-based prediction are shown in Supplementary figure 7 and Supplementary figure 8, respectively. As we explained above, the EUTOS score is calculated by two variables, spleen size and basophil ratio. The score is expressed by a sum of each variable's monotonically increasing linear functions. It means that the EUTOS score is designed so that patients with smaller variable values tend to be classified as "low risk" corresponding to "prediction as DMR". Patients with larger variable values tend to be classified as "high risk" corresponding to "prediction as non-DMR". However, the average spleen size of FN patient(s) (patients who achieved DMR but were predicted as non-DMR) is larger than that of TN patients (patients who did not achieve DMR and were correctly predicted as non-DMR) as shown in Supplementary figure 7 (**a**-**c**). The same tendency was confirmed in the case of basophil ratio except for MR4.0 (Supplementary figure 7 **a**-**c**). Although the average spleen size of FP patients was larger than that of TP patients (Supplementary figure 7 **a**-**c**), the distribution of FP patients and that of TP patients are not separated (Supplementary figure 7 **d**-**f**). This tendency was also confirmed in the case of basophil ratio. As above, actual observations of DMR and the design of EUTOS scores showed some discordance. Therefore, the EUTOS score-based prediction did not work well.

We also performed the same analysis to the results of the ELTS score-based predictions (Supplementary figure 8). As indicated above, the ELTS score is defined by four variables, age in completed years, spleen size, blasts percentage in peripheral blood, and platelet count. The ELTS score is designed so that a patient whose these variable values are large tends to be predicted as "high risk" or "intermediate risk", which corresponds to "prediction as non-DMR" in this study. However, in the cases of age and platelet count, FN patients had larger average values compared to TN patients (Supplementary figure 8 **a**-**c**). In the cases of spleen size and blasts ratio, although the average values of FN patients were smaller than those of TN patients, the distributions of FN patients and those of TN patients were not separated (Supplementary figure 8 **d**-**f**). As above, similar to the EUTOS score, actual observations of DMR and the design of ELTS scores showed some discordance. Therefore, the ELTS score-based prediction did not also work well.

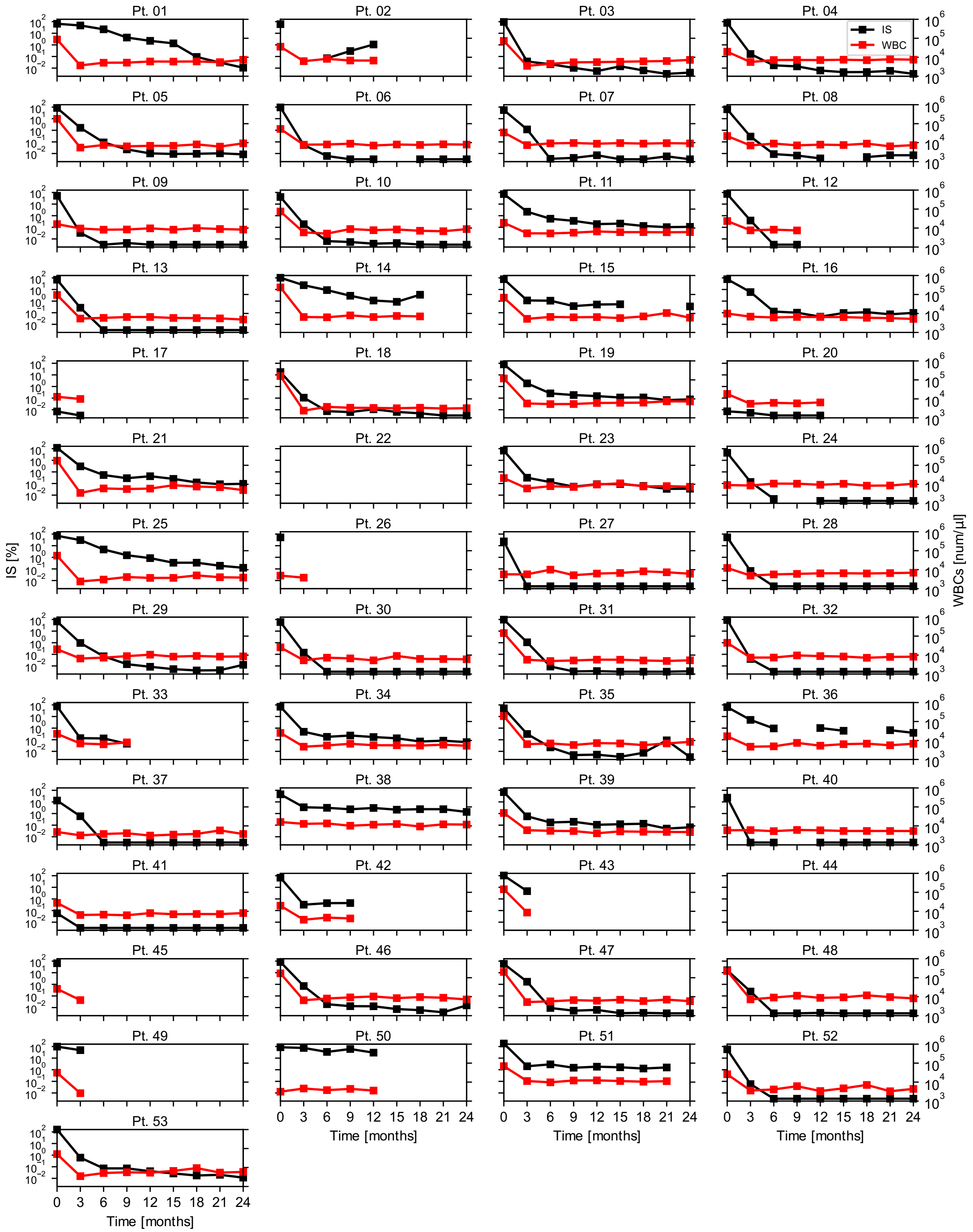
Pt. 01
Pt. 02
Pt. 03
Pt. 04
IS
WBC
Pt. 05
Pt. 06
Pt. 07
Pt. 08
Pt. 09
Pt. 10
Pt. 11
Pt. 12
Pt. 13
Pt. 14
Pt. 15
Pt. 16
Pt. 17
Pt. 18
Pt. 19
Pt. 20
Pt. 21
Pt. 22
Pt. 23
Pt. 24
Pt. 25
Pt. 26
Pt. 27
Pt. 28
Pt. 29
Pt. 30
Pt. 31
Pt. 32
Pt. 33
Pt. 34
Pt. 35
Pt. 36
Pt. 37
Pt. 38
Pt. 39
Pt. 40
Pt. 41
Pt. 42
Pt. 43
Pt. 44
Pt. 45
Pt. 46
Pt. 47
Pt. 48
Pt. 49
Pt. 50
Pt. 51
Pt. 52
Pt. 53
IS [%]
WBCs [num/µl]
Time [months]

Supplementary figure 1. Measurement data for CML patients. Our prediction method used the time-series data for the white blood cell (WBC) counts and the ratio of *BCR-ABL1* mRNA to *ABL1* mRNA on the international scale (IS). The dataset consists of data from 53 patients. However, for 21 patients, the WBC count and/or IS data were incomplete at a certain time point. Thus, in this study, we used only the remaining 32 patient data.

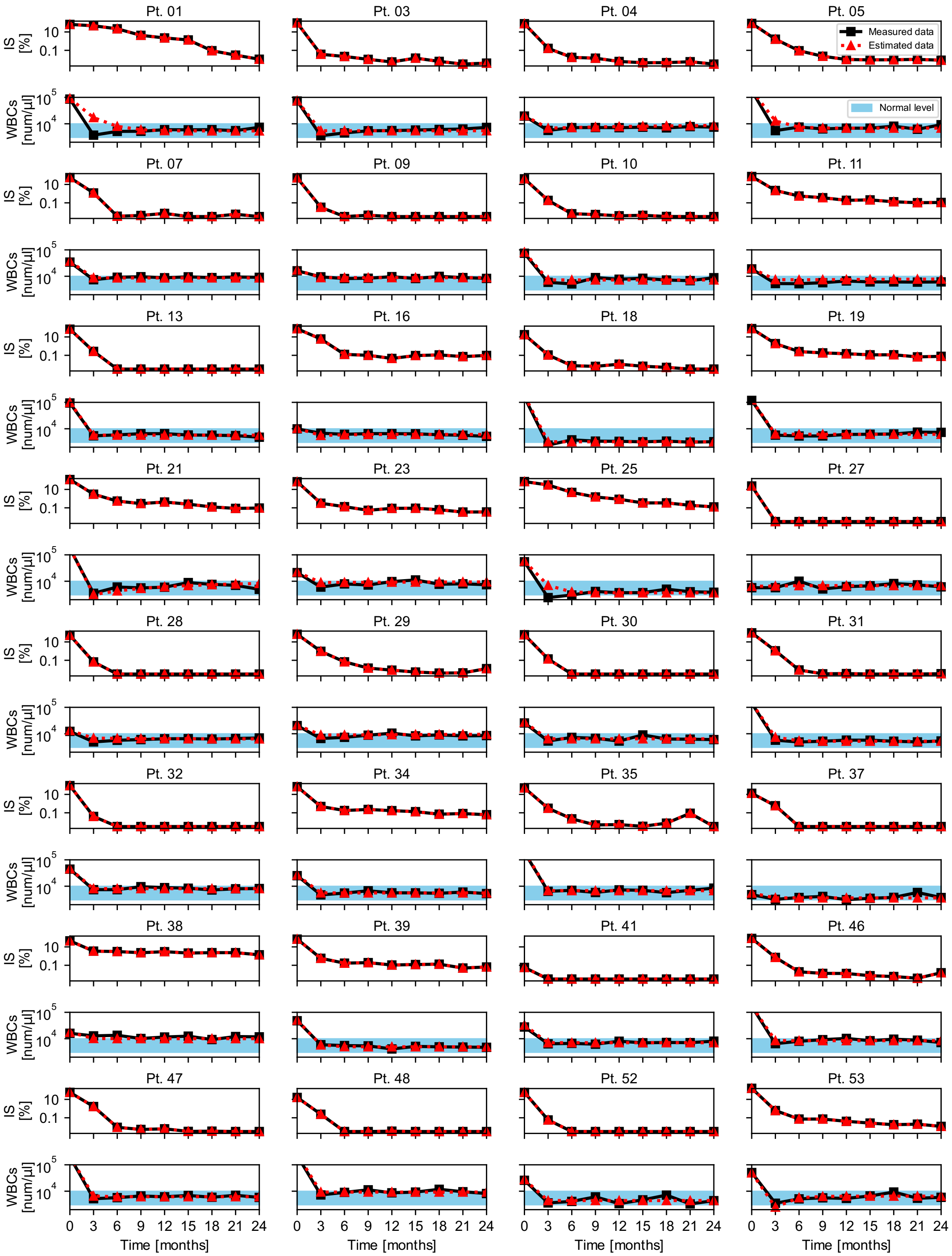

Supplementary figure 2. Our CML model approximated all measurement data. Each black solid line represents the WBC and IS measurement data for each patient. Each red dashed line

represents the estimated dynamics based on our proposed model. Our model approximated all measurement data sufficiently.

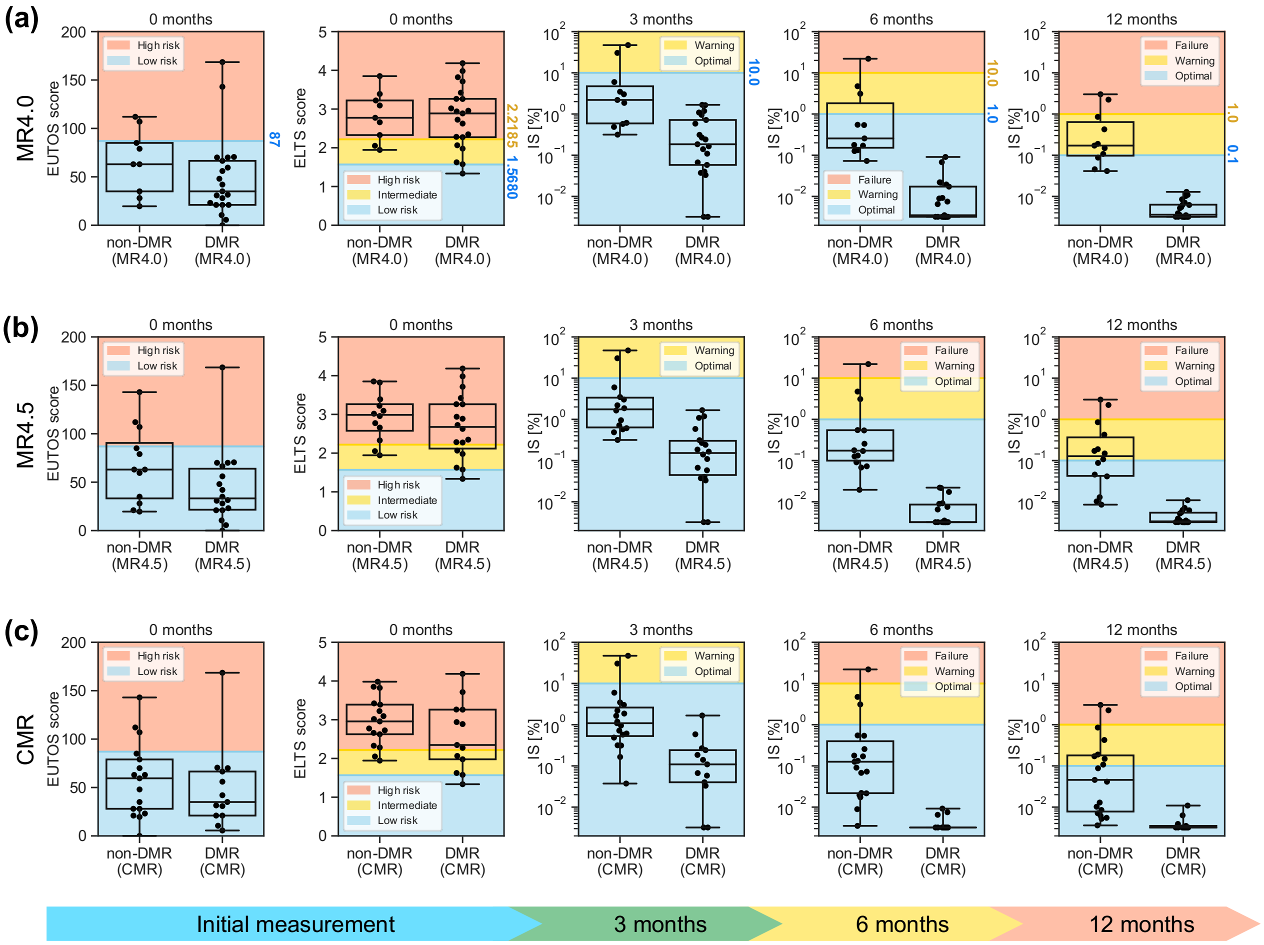

Supplementary figure 3. Effectiveness of the EUTOS score, ELTS score, and European LeukemiaNet guideline criteria for MR4.0, MR4.5, and CMR. (**a-c**) Based on the approach described in Fig.1, we show the distributions of the EUTOS score, the ELTS score, and the European LeukemiaNet (ELN) guideline for (**a**) MR4.0, (**b**) MR4.5, and (**c**) CMR (see Methods in the main text for the definitions of MR4.0, MR4.5, and CMR). In most cases except for the guideline at 12 months for MR4.0 (the right top panel), these three approaches did not accurately predict non-MR4.0, non-MR4.5, and non-CMR patients.

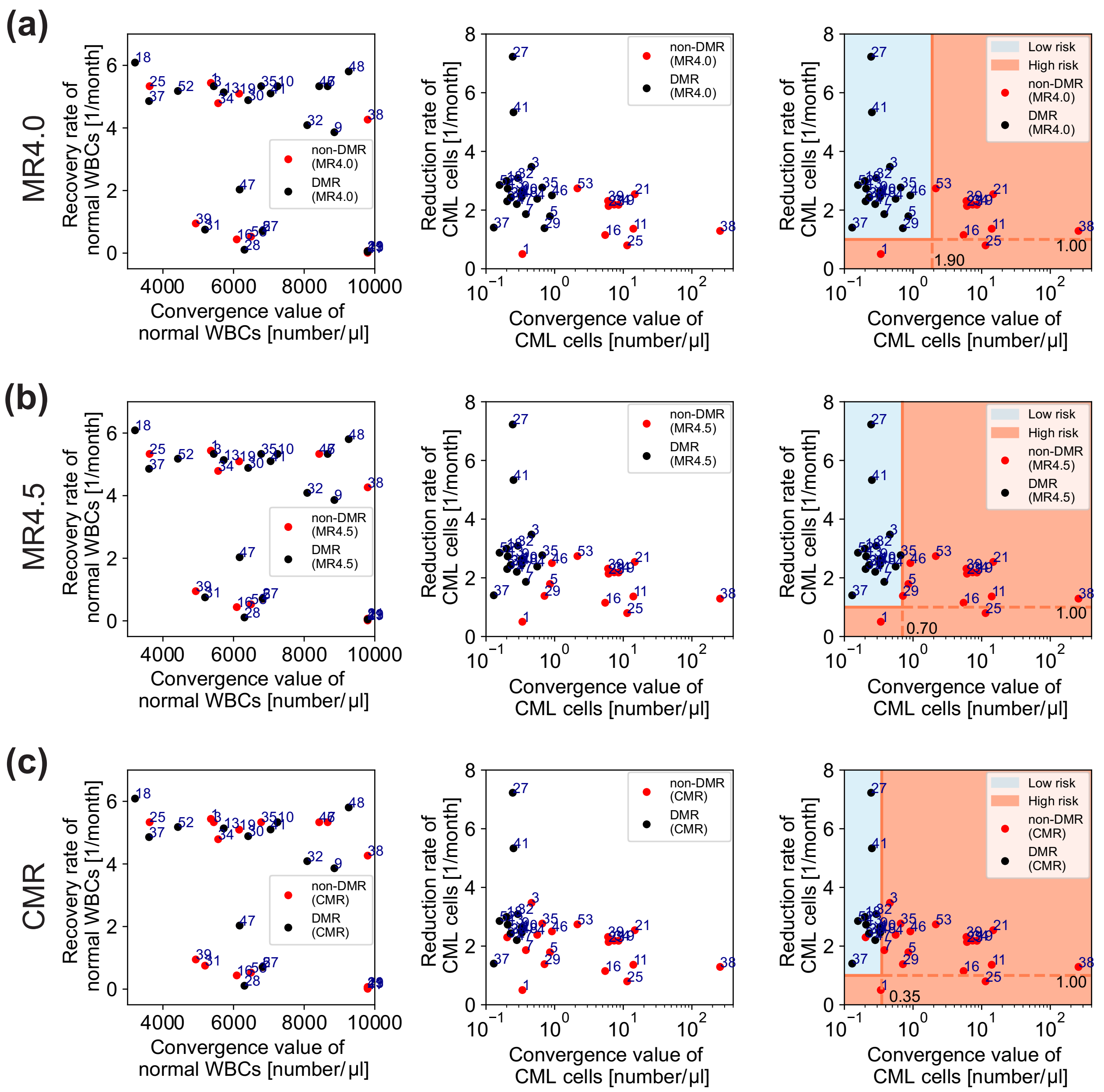

Supplementary figure 4. The classification of MR4.5 and CMR patients required the lower CML cell convergence value than that of MR4.0 patients. (**a**-**c**) Distributions of the reduction rates and convergence values for (left) normal WBCs, (middle) CML cells, and (right) the classification. The labels of these figures are (**a**) MR4.0, (**b**) MR4.5, and (**c**) CMR.

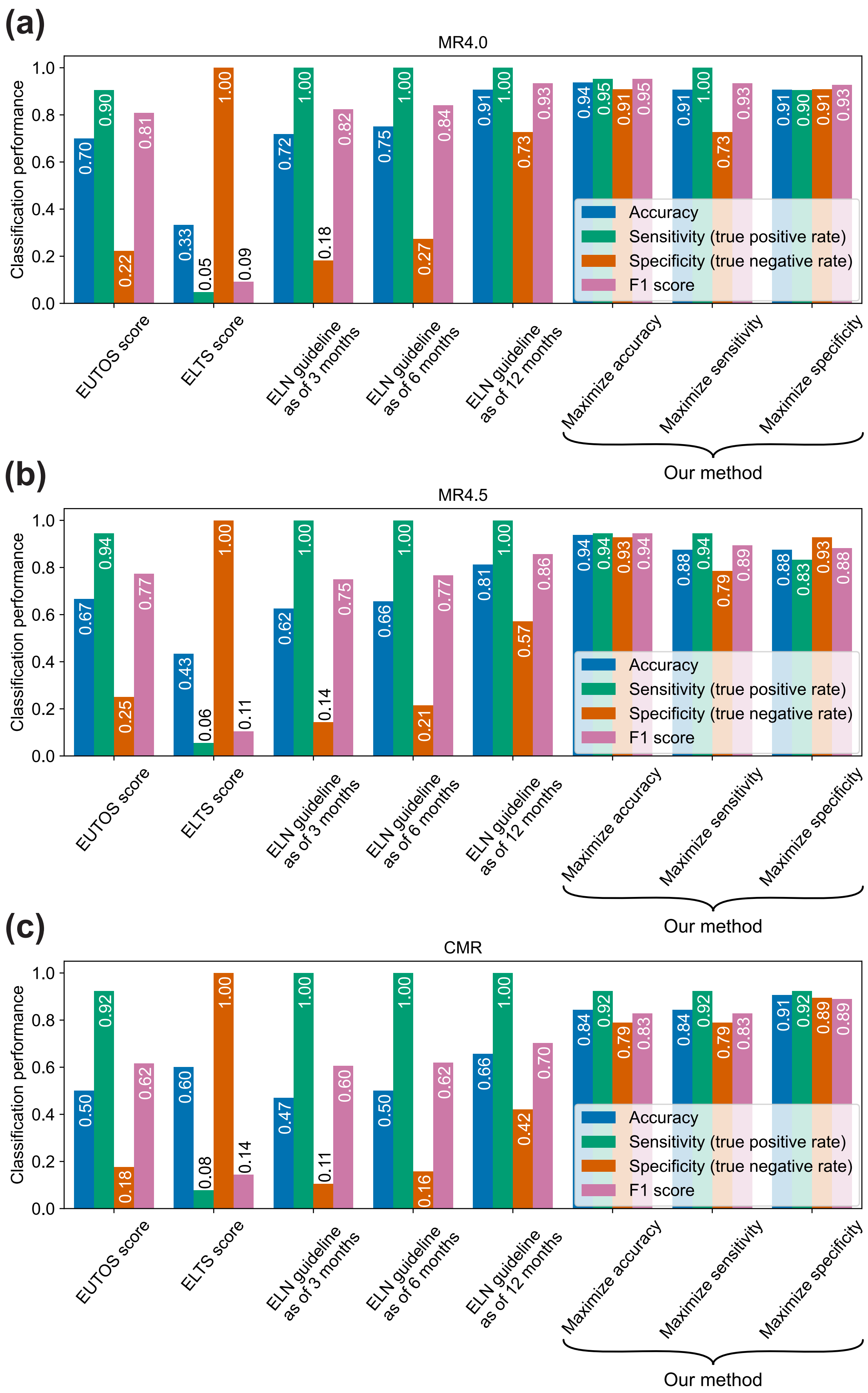
(a)
MR4.0
Classification performance
1.0
0.8
0.6
0.4
0.2
0.0
0.70
0.90
0.22
0.81
0.33
0.05
1.00
0.09
0.72
1.00
0.18
0.82
0.75
1.00
0.27
0.84
0.91
1.00
0.73
0.93
0.94
0.95
0.91
0.95
0.91
1.00
0.73
0.93
0.91
0.90
0.91
0.93
Accuracy
Sensitivity (true positive rate)
Specificity (true negative rate)
F1 score
EUTOS score
ELTS score
ELN guideline as of 3 months
ELN guideline as of 6 months
ELN guideline as of 12 months
Maximize accuracy
Maximize sensitivity
Maximize specificity
Our method
(b)
MR4.5
Classification performance
1.0
0.8
0.6
0.4
0.2
0.0
0.67
0.94
0.25
0.77
0.43
0.06
1.00
0.11
0.62
1.00
0.14
0.75
0.66
1.00
0.21
0.77
0.81
1.00
0.57
0.86
0.94
0.94
0.93
0.94
0.88
0.94
0.79
0.89
0.88
0.83
0.93
0.88
Accuracy
Sensitivity (true positive rate)
Specificity (true negative rate)
F1 score
EUTOS score
ELTS score
ELN guideline as of 3 months
ELN guideline as of 6 months
ELN guideline as of 12 months
Maximize accuracy
Maximize sensitivity
Maximize specificity
Our method
(c)
CMR
Classification performance
1.0
0.8
0.6
0.4
0.2
0.0
0.50
0.92
0.18
0.62
0.60
0.08
1.00
0.14
0.47
1.00
0.11
0.60
0.50
1.00
0.16
0.62
0.66
1.00
0.42
0.70
0.84
0.92
0.79
0.83
0.84
0.92
0.79
0.83
0.91
0.92
0.89
0.89
Accuracy
Sensitivity (true positive rate)
Specificity (true negative rate)
F1 score
EUTOS score
ELTS score
ELN guideline as of 3 months
ELN guideline as of 6 months
ELN guideline as of 12 months
Maximize accuracy
Maximize sensitivity
Maximize specificity
Our method

Supplementary figure 5. Our prediction method can also be applied to MR4.0, MR4.5 and CMR. (**a**-**c**) Using the approach described in Fig. 4, we obtained the accuracy, sensitivity, specificity, and F1 score performance for the EUTOS score, the ELTS score, the ELN guidelines, and our proposed method. The labels of these panels are (**a**) MR4.0, (**b**) MR4.5, and (**c**) CMR. The performance of our method preserved its performance even in CMR cases.

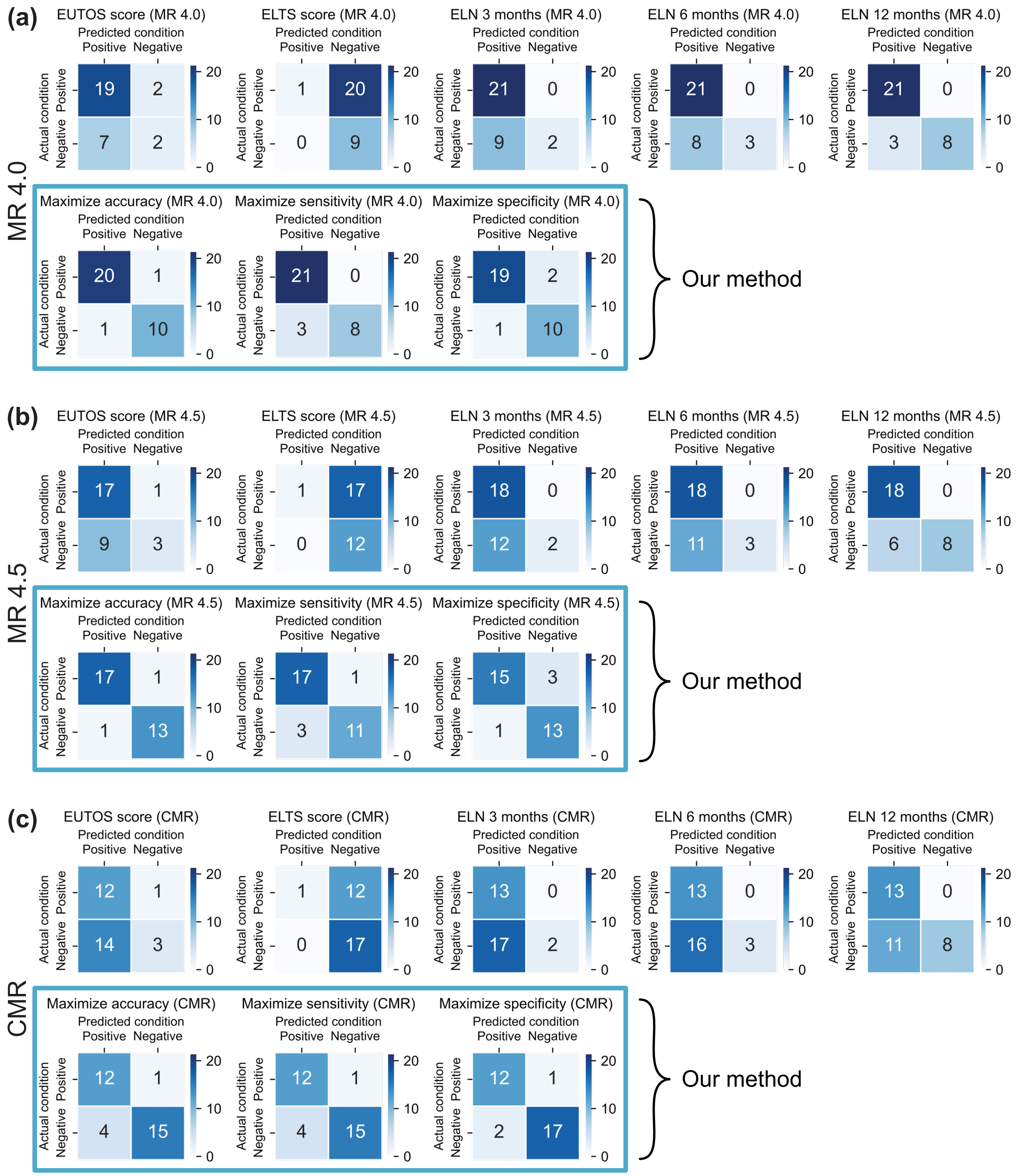

Supplementary figure 6. Confusion matrices of DMR prediction based on our prediction method and the criteria of the current scoring systems and guidelines. (a-c) Each confusion matrix indicates correctness of the prediction results based on each method/scoring

system/guideline. A number indicated in a cell of the matrices is the number of patients corresponding to the predicted and actual conditions. We note that the classification performance scores (accuracy, sensitivity, specificity, and F1 score) shown in Fig.4 and Supplementary figure 5, were calculated from these matrices. The definition of DMR is set at (**a**) MR4.0, (**b**) MR4.5, and (**c**) CMR.

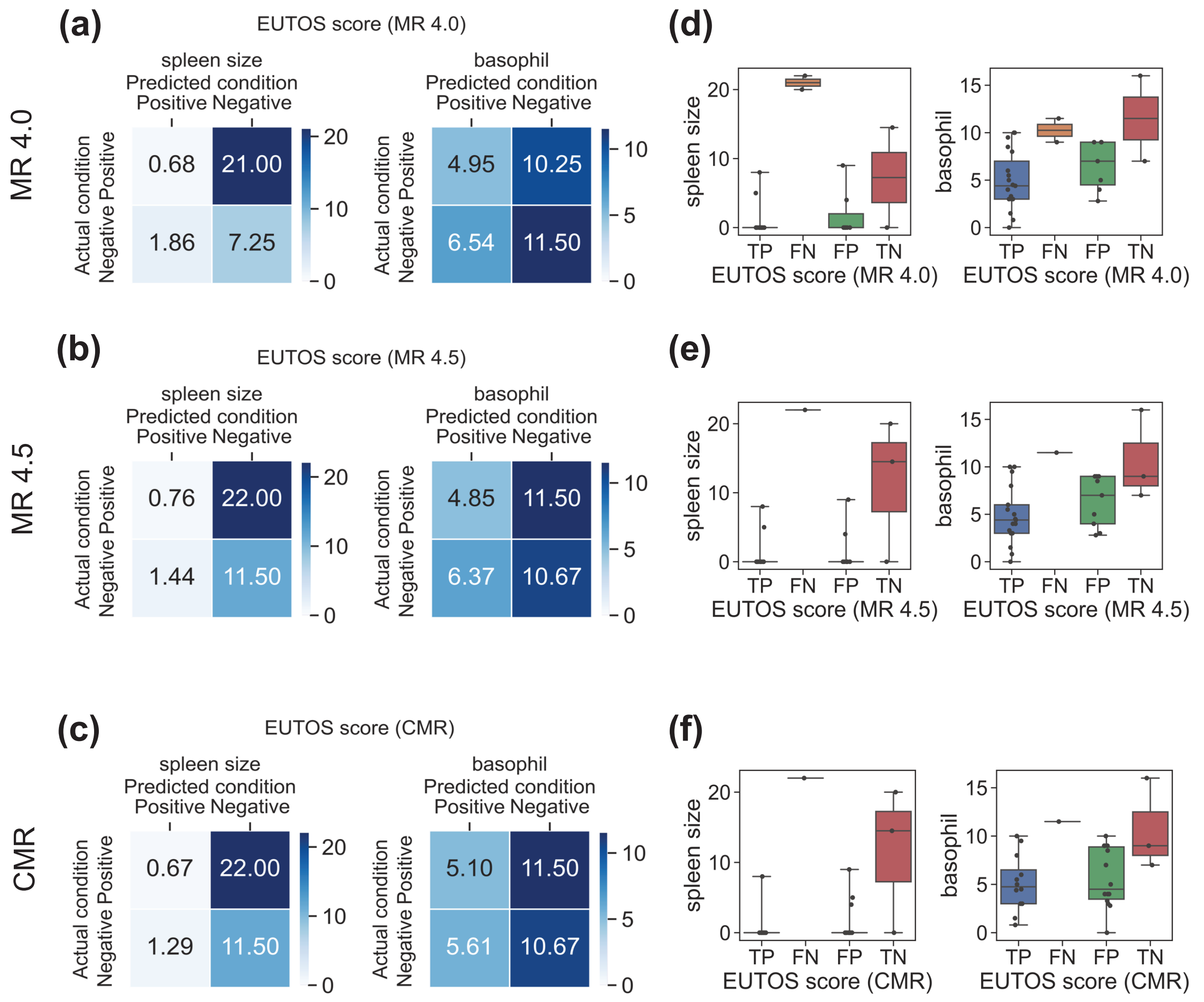

Supplementary figure 7. Patient characteristics based on the results of the EUTOS score-based DMR prediction. (**a-c**) Average values of spleen size (left) and basophil ratio (right) among the patients classified into four subsets based on EUTOS score, corresponding to each top-left matrix of Supplementary figure 6(**a-c**). (**d-e**) Distributions of spleen size (left) and basophil ratio (right) for the four subsets of patients. We note that TP, FN, FP, and TN correspond to top-left, top-right, bottom-left, and bottom-right of the matrices of (**a-c**), respectively. Each dot indicates each patient value. The top and bottom bars of the boxes indicate the maximum and minimum

values, respectively. The top, center, and bottom lines are the upper quartiles, medians, and lower quartiles, respectively. The definition of DMR is set at (**a**, **d**) MR4.0, (**b**, **e**) MR4.5, and (**c**, **f**) CMR.

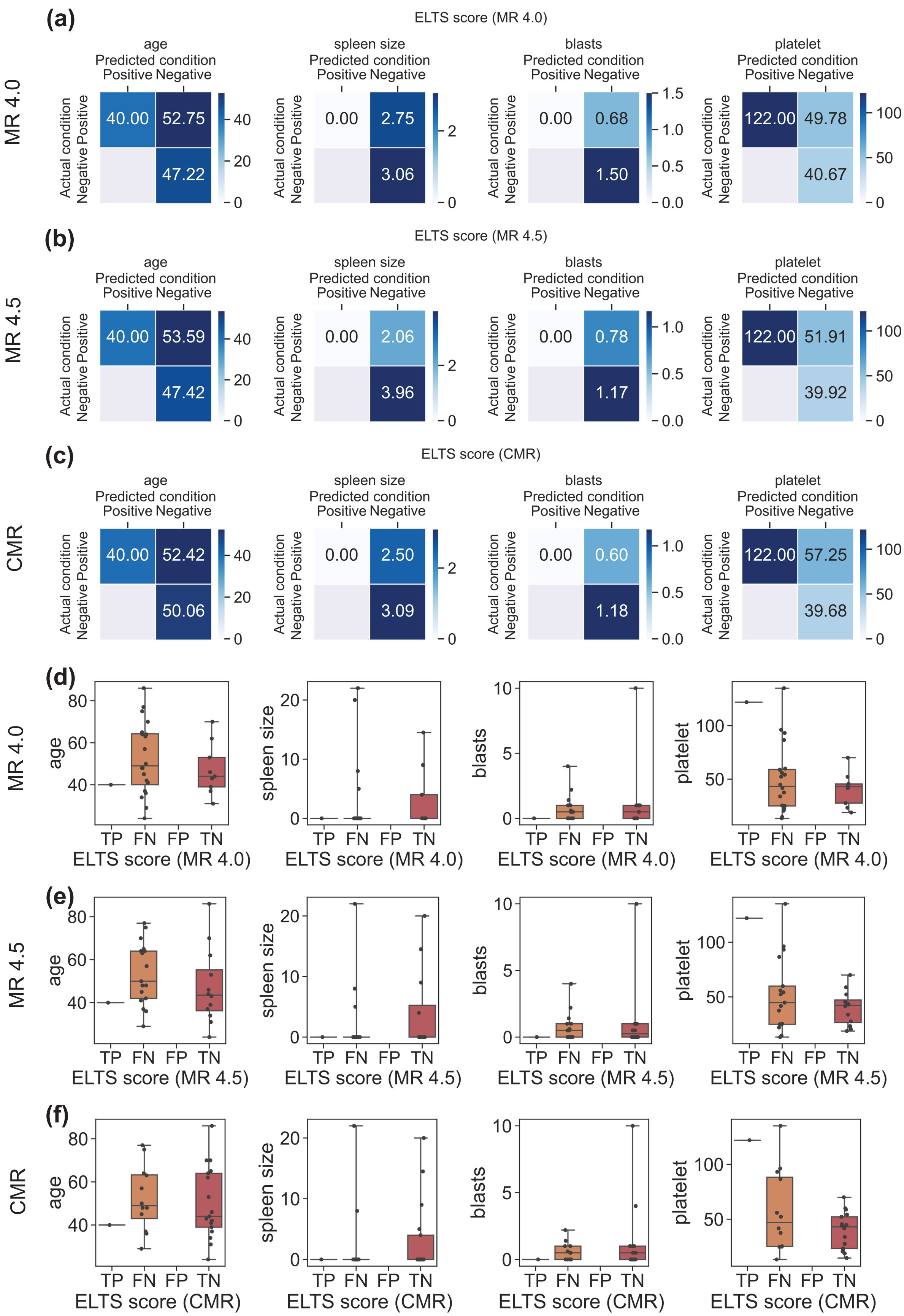
(a)
ELTS score (MR 4.0)
MR 4.0
age
spleen size
blasts
platelet
Predicted condition
Positive Negative
Actual condition
Negative Positive
40.00
52.75
47.22
0.00
2.75
3.06
0.00
0.68
1.50
122.00
49.78
40.67
(b)
ELTS score (MR 4.5)
MR 4.5
40.00
53.59
47.42
0.00
2.06
3.96
0.00
0.78
1.17
122.00
51.91
39.92
(c)
ELTS score (CMR)
CMR
40.00
52.42
50.06
0.00
2.50
3.09
0.00
0.60
1.18
122.00
57.25
39.68
(d)
(e)
(f)
TP FN FP TN
ELTS score (MR 4.0)
ELTS score (MR 4.5)
ELTS score (CMR)

Supplementary figure 8. Patient characteristics based on the results of the ELTS score-based DMR prediction. (**a**-**c**) Average values of age in completed years (left), spleen size below costal margin (the second left), percentage of blasts in peripheral blood (the third left) and platelet count (right) among the patients classified into four subsets based on ELTS score, corresponding to each second left matrix on the upper row of Supplementary figure 6(**a**-**c**). (**d**-**e**) Distributions of age in completed years (left), spleen size below costal margin (the second left), percentage of blasts in peripheral blood (the third left) and platelet count (right) for the four subsets of patients. The definitions of TP, FN, FP, and TN are the same as those in Supplementary figure 7. The definitions of the bars of the boxes, lines, and dots used in the boxplots (**d**-**f**) are also the same as those in Supplementary figure 7(**d**-**e**). The definition of DMR is set at (**a**, **d**) MR4.0, (**b**, **e**) MR4.5, and (**c**, **f**) CMR.

Supplementary data 1. (separate file)

Time-series data of the IS values, the WBC counts, and other values for the EUTOS score and the ELTS score are described in data.csv. Note that the data contain missing values. See "Detailed information of the N-road trial" in the main text for how we dealt with the missing values.
